# Supplementary material for: Genome-wide CRISPR Screens in T Helper Cells Reveal Pervasive Crosstalk between Activation and Differentiation
Source: Cell. 2019 Feb 7;176(4):882–896.e18. doi: 10.1016/j.cell.2018.11.044 (PMC6370901; doi:10.1016/j.cell.2018.11.044)
Supplement: Data S2. Processed Data from All the Steps of the Analysis, Related to Figure 1 [file mmc2.zip › supplemental data/motif analysis/Stat6_SRX021632_homer/homerResults/motif12.similar.html]

motif12

## Information for motif12

G
T
C
A
A
C
T
G
C
G
T
A
A
C
G
T
C
G
T
A
C
G
T
A
T
C
A
G
T
G
C
A
  
Reverse Opposite:  

A
C
G
T
A
G
T
C
A
C
G
T
A
C
G
T
C
G
T
A
A
C
G
T
A
G
T
C
C
A
G
T
  

|  |  |
| --- | --- |
| p-value: | 1e-191 |
| log p-value: | -4.400e+02 |
| Information Content per bp: | 1.857 |
| Number of Target Sequences with motif | 3389.0 |
| Percentage of Target Sequences with motif | 13.41% |
| Number of Background Sequences with motif | 1916.3 |
| Percentage of Background Sequences with motif | 7.94% |
| Average Position of motif in Targets | 101.1 +/- 54.1bp |
| Average Position of motif in Background | 102.5 +/- 60.6bp |
| Strand Bias (log2 ratio + to - strand density) | 0.0 |
| Multiplicity (# of sites on avg that occur together) | 1.13 |
| Motif File: | file (matrix) reverse opposite |

### Similar de novo motifs found

|  |  |  |  |  |  |  |  |
| --- | --- | --- | --- | --- | --- | --- | --- |
| Rank | Match Score | Redundant Motif | P-value | log P-value | % of Targets | % of Background | Motif file |
| 1 | 0.939 | C T A G G T C A A G C T A G T C A C G T A C G T C G T A A C G T A G T C C G A T | 1e-181 | -417.480190 | 9.04% | 4.74% | motif file (matrix) |
| 2 | 0.632 | A G C T A C T G A G T C G T A C G T C A A C G T A G T C A C G T | 1e-176 | -407.215111 | 11.47% | 6.60% | motif file (matrix) |
| 3 | 0.792 | A G T C G C A T C A T G G T C A A C G T A T G C G A C T A G C T G T C A C A G T A G T C G C A T | 1e-71 | -164.562977 | 3.31% | 1.67% | motif file (matrix) |
| 4 | 0.656 | C G T A T A C G T G C A C G A T T G A C C G T A A T C G G C T A C G A T T C G A T C G A A T G C | 1e-66 | -153.165795 | 0.25% | 0.01% | motif file (matrix) |
